# *TNFR1* single nucleotide polymorphisms are not associated with cervical HPV-induced pre-malignant lesion but regulate *in situ* cervical TNFR1 expression

**DOI:** 10.18632/oncotarget.26627

**Published:** 2019-01-29

**Authors:** Natália Pereira da Rocha, Elyzabeth Avvad-Portari, Fábio Russomano, Eric Henrique Roma, Amanda Chaves Pinto, Evandro Klumb, Jacyara Macedo, Ana Teresa Gomes Fernandes, Maria da Glória Bonecini-Almeida

**Affiliations:** ^1^ Laboratory of Immunology and Immunogenetics in Infectious Diseases, Evandro Chagas National Institute of Infectious Diseases, Oswaldo Cruz Foundation, Rio de Janeiro, Brazil; ^2^ Department of Pathologic Anatomy, Fernandes Figueira Woman, Child and Adolescent National Institute, Oswaldo Cruz Foundation, Rio de Janeiro, Brazil; ^3^ Women′s Health Care Area, Fernandes Figueira Woman, Child and Adolescent National Institute, Oswaldo Cruz Foundation, Rio de Janeiro, Brazil; ^4^ Department of Biochemistry, State University of Rio de Janeiro, Rio de Janeiro, Brazil; ^5^ Department of Rheumatology, State University of Rio de Janeiro, Rio de Janeiro, Brazil

**Keywords:** HPV, cervical lesions, in situ TNFR1, TNFR1 SNPs

## Abstract

TNF-α is involved in HPV infection control by triggering cell signaling through binding in specific receptors TNFR1 and TNFR2. Genetic polymorphisms in these receptors may influence TNF-α signaling. Herein, we investigated *TNFR1* rs767455 and rs2234649 single nucleotide polymorphisms, and TNFR1 protein expression in cervical squamous intraepithelial lesions (SIL) to identify their role in cervical pre-malignant development. SIL patients (*n* = 179) and healthy volunteers (*n* = 227) were enrolled for TNFR1 genotyping analysis by PCR-RFLP in blood samples and TNFR1 protein expression in cervical tissue by immunohistochemistry. No statistical differences regard genotypes and allelic frequencies for both polymorphisms were observed. Cervical TNFR1-expressing cells were rare in epithelium and basal layer regardless the groups. However, a progressive increase in infiltrating cells was observed in the stromal area, mainly in high SIL (HSIL) group compared to low SIL (LSIL, *p* < 0.001) and control (*p* < 0.001) groups. TNFR1-expressing cells frequency was higher in TNFR1 *rs767455AG/GG* (*p* < 0.001), and in *rs2234649AA* (*p* < 0.001) genotypes carries in HSIL subgroup. These data indicated that TNFR1-expression is abrogated in cervical epithelium, where HPV-induced pre-malignant lesion occurs, increasing its frequency in inflammatory cells in stroma, and is genetically controlled by *TNFR1 rs767455AG*/GG and *rs234649AA* genotypes. These biomarkers may be useful to identify cervical precancerous lesions progression.

## INTRODUCTION

Cervical cancer is the fourth common cancer among women around the world, and human papillomavirus (HPV) infection is the primary cause of this disease [1]. HPV prevalence has been steadily increasing in the last decades, and became one of the most prevalent sexually transmitted infections in the world, with high prevalence in Africa (21.4%), Western Europe (21.45%) and Latin America (16.1%) [2–4]. In Brazil, the prevalence ranged from 10.4 to 24.5% [5], although, a recent surveillance conducted by Brazilian Ministry of Health showed that 53,6% of young person from 16 to 26 years old were infected and 35,2% had high risk to HPV infection [6].

Cervical HPV infection is characterized by a chronic inflammation that induces intense immunological mechanisms including phagocytosis and cellular immunity, also triggering the production of many pro-inflammatory cytokines by infiltrating lymphocytes, macrophages and infected keratinocytes [7]. Previous results from our group described the presence of lymphocytic infiltrate and IL-6, IFN-γ and TNF-α expressing cells in cervical lesions [8].

TNF-α is one of the main mediators of inflammation in the skin and mucosae. When produced by tumor and inflammatory cells in the tumor microenvironment, can promote the survival of malignant cells by inducing gene encoding anti-apoptotic molecules in a NFkB-dependent manner [9]. This cytokine is also able to stimulate the production of genotoxic molecules, such as nitric oxide and reactive oxygen species that cause DNA damage and mutations, and thus contribute to tumor initiation, progression, angiogenesis and metastasis [10]. TNF-α is involved either directly or indirectly in control of HPV infection. TNF-α is constitutively produced in HPV-harboring cervical keratinocytes [11] and in cervical lesions, where TNF-α was found mainly in macrophage-like cells in stroma [8]. In fact, it has been shown that HPV16-positive cervical cell lines have increased levels of TNF-α mRNA compared to HPV-negative ones [12]. E6 protein from high-risk HPV16 mediates p53 degradation and induces cell transformation. Transfection of HPV16 E6 into TNF-sensitive LM cell line (a mouse fibroblast) protects E6 expressing cells from TNF-induced apoptosis in a p53 independent manner [13]. HPV16 E6 oncoprotein binds directly to TNFR1, requiring the same C-terminal portion of TNFR1 as does TNFR1 associated death domain (TRADD) and E6 decreases TNFR1/TRADD association [14]. On the other hand, while E7 does not subvert signaling by TNFR1, pro-caspase 8 activation is decreased in E7- expressing fibroblasts. In addition, E7 also provides some protection from apoptosis caused by stimulation of the TNFR1- related cytokine receptor Fas, inducing much slower apoptosis in this cell type [15]. Recent data demonstrated that TNF-α was downregulated at both mRNA and protein levels in cervical cancer and in CIN cases compared to controls. Moreover, TNF-α expression was correlated with insufficient modulation of IFN-γ and inversely correlated with HPV16 E6 and E7 transcripts in cervical cancer cases [16].

TNF-α exerts its biological activity at the cellular level, through its binding with the transmembrane receptors, TNFR1 [55kDa] and TNFR2 [75kDa]. Both are single transmembrane glycoproteins which can induce cell apoptosis or cell survival; however, the majority of signals are transmitted by TNFR1. TNFR1 is expressed in almost all cell types and has pleiotropic effects, acting on both NF-kB activation, and apoptosis induction. This receptor is the main mediator of TNF signaling pathways, affecting the binding of TNF-α in the membrane [17]. A growing number of genetic studies have identified several single nucleotide polymorphisms (SNPs) in the *TNFR1A* gene as susceptibility or predictive markers of multifactorial inflammatory disorders [18–22] and cancer [23–28]. One of the functional consequences of these variants could be linked to regulation of gene expression [29]. However, to our knowledge, there are no published studies regarding the relationship between SNPs in the *TNFR1A* gene, the *in situ* protein expression, and the cervical lesion induced by HPV-infection among Brazilian population. In this study, we selected *TNFR1* rs767455 *(+36A/G)* and *TNFR1* rs2234649 *(-383A/C)* SNPs and determined their genotypes in a relatively large sample size. We then examined the correlation of these SNPs with the risk for cervical lesion development, and the effect of these observed variations in association with risk factors and TNFR1 expressing cells.

## RESULTS

### Characteristics of study population

A total of 406 women were enrolled for the genotyping study from clinical sites. From those, 179 SIL patients, classified as either LSIL (*n* = 78) or HSIL (*n* = 101), and 227 volunteers showing no lesions upon gynecological evaluation were enrolled. Table 1 describes clinical and demographics data on each studied group. Social and environmental data from SIL and control groups were identified such as age, self-determined ethnicity, tobacco use, age at first sexual intercourse, menarche and number of pregnancies. No significant difference was found between SIL and control group in all co-variables described, except age (37.70 ± 10.75 and 34.78 ± 10.62 years old, respectively, *p* < 0.01) and age at first sexual intercourse (*p* < 0.001). Volunteers from control group were younger than SIL patients and HSIL subgroup (*p* < 0.01). Interestingly, patients with HSIL were more likely to use tobacco and had first sexual intercourse earlier when compared to control group, *p* = 0.015 and *p* < 0.001, respectively. In the LSIL and HSIL subgroups, the average age was 35.95 ± 10.72 and 39.05 ± 10.63 years old, respectively, and co-variables were not significantly different in both subgroups (data not shown).

**Table 1:** Clinical and environmental data

	Control	SIL	LSIL	HSIL	p^*^	p^**^	p^***^
	*n* = 227(%)	*n* = 179(%)	*n* = 78(%)	*n* = 101(%)			
*Age years (mean ± SD)*	34.78 ± 10.62	37.70 ± 10.75	35.95 ± 10.72	39.05 ± 10.63	<0.01^a^	>0.05^a^	<0.01a
*Ethinicity (n,%)*							
White	87 (38)	61 (34)	31 (40)	30 (30)	0.01^b^	0.148^b^	0.006^b^
Afro-Brazilian	133 (59)	113 (63)	46 (59)	67 (66)			
Indigen	-	2 (1)	1 (1)	1 (1)			
Asian-Brazilian	-	3 (2)	-	3 (3)			
No determinated	7 (3)	-	-	-			
*Tobacco use (n,%)*							
Yes	80 (35)	99 (55)	37 (47)	62 (61)	0.08^c^	0.86^c^	0.015^c^
No	93 (41)	80 (45)	41 (53)	39 (39)			
Unknown	54 (24)	-	-	-			
*Age at first sexual intercourse (average ± SD)*	18.10 ± 3.50	17.27 ± 3.70	17.72 ± 4.09	16.92 ± 3.35	<0.001^a^	>0.05^a^	<0.001^ba^
*Menarche (average ± SD)*	12.73 ± 1.73	12.66 ± 1.62	12.65 ± 1.80	12.66 ± 1.49	>0.05^a^	>0.05^a^	>0.05^a^
*Number of pregnancies (average ± SD)*	2.31 ± 1.72	2.55 ± 1.88	2.17 ± 1.83	2.85 ± 1.82	>0.05^a^	>0.05^a^	>0.05^a^

SIL, Squamous Intraepithelial Lesions; LSIL, Low SIL; HSIL, High SIL.

^*^p value comparing SIL with control, ^**^p value comparing LSIL with control, ^***^p value comparing HSIL with control.

^a^Unpaired *t* test, Mann–Whitney.

^b^Two way contingency table, from χ^2^ test.

^c^Two sided, from χ^2^ test.

### *TNFR1 rs767455A>G* and *TNFR1 rs2234649A>C* gene frequency and their associations with cervical lesions

The genotypes and alleles distribution of *TNFR1 rs767455* and *rs2234649* in SIL patients and control group are summarized in Table 2. The observed genotype frequencies of these SNPs agreed with Hardy-Weinberg equilibrium either in the case and control groups in *rs767455* (*p* = 0.5 and 0.2, respectively) and *rs2234649* (*p* = 0.1 and 0.06, respectively). No associations in genotype and allelic frequencies of *TNFR1 rs767455* and *TNFR1 rs2234649* polymorphisms were observed between SIL and control groups (Table 2), even when the SIL group was stratified into LSIL and HSIL subgroups (Table 3). No association was identified between LSIL and HSIL subgroups (p>0.05), in any genetic model tested (data not shown). There was no significant difference when the OR was adjusted for univariate model including age, ethnicity, tobacco use, age at first sexual intercourse, menarche, and number of pregnancies or in multivariate analysis (data not shown). These variables did not change the risk for SIL development. These results demonstrated no association of *TNFR1 rs767455* and *rs2234649* polymorphisms with cervical lesion progression induced by HPV-infection, in any genetic model tested.

**Table 2:** Logistic regression analysis of associations between TNFR1 *rs767455 A>G* and TNFR1 *rs2234649 A>C* polymorphisms and risk of squamous intraepithelial lesions

Polymorphisms	Control	SIL	*p*	OR
	*n* = 227 (%)	*n* = 179 (%)		(95% CI)
*TNFR1 rs767455 A>G*				
AA	101 (44)	79 (44)	0.55^a^	1
GA	106 (47)	77 (43)		0.92
GG	20 (9)	23 (13)		1.47
AA	101 (44)	79 (44)	0.94^b^	1
GA+GG	126 (56)	100 (56)		0.98 (0.66-1.46)
GG	20 (9)	23 (13)	0.18^b^	1
AA+GA	207 (91)	156 (87)		1.52 (0.80-2.87)
*Alleles*				
A	308 (68)	235 (66)	0.50^b^	1
G	146 (32)	123 (34)		0.90 (0.67-1.21)
*TNFR1 rs2234649 A>C*				
AA	175 (77)	135 (75)	0.77^a^	1
AC	45 (20)	38 (21)		1.09
CC	7 (3)	6 (4)		1.11
AA	175 (77)	135 (75)	0.69^b^	1
AC+CC	52 (23)	44 (25)		0.91 (0.57-1.44)
CC	7 (3)	6 (4)	0.87^b^	1
AA+AC	220 (97)	173 (96)		1.09 (0.35-3.30)
*Alleles*				
A	392 (87)	308 (86)	0.71^b^	1
C	59 (13)	50 (14)		0.92 (0.61-1.39)

SIL squamous intraepithelial lesion; OR, odds ratio.

^a^*p* value, two sided, from χ^2^ test for trend.

^b^*p* value, two sided, from χ^2^ test.

**Table 3:** Logistic regression analysis of associations between TNFR1 rs767455 A>G and TNFR1 rs2234649 A>C polymorphisms and risk of squamous intraepithelial lesions progression

		SIL (*n* = 179)					
	Control	LSIL	p^*^	OR	HSIL	p^**^	OR
	*n* = 227 (%)	*n* = 78 (%)		(95% CI)	*n* = 101 (%)		(95% CI)
TNFR1 rs767455 A>G							
AA	101 (44)	30 (38)	0.34a	1	49 (48)	0.96a	1
AG	106 (47)	39 (50)		1.23	38 (38)		0.73
GG	20 (9)	9 (12)		1.51	14 (14)		1.43
AA	101 (44)	30 (38)	0.35b	1	49 (48)	0.49b	1
AG+GG	126 (56)	48 (62)		0.77 (0.46–1.31)	52 (52)		1.17 (0.80–3.44)
GG	20 (9)	9 (12)	0.47b	1	14 (14)	0.16b	1
AG+AA	207 (91)	69 (88)		1.35 (0.58–3.10)	87 (86)		1.66 (0.73–1.88)
Alleles							
A	308 (68)	99 (63)	0.27b	1	136 (67)	0.89b	1
G	146 (32)	57 (37)		0.80 (0.55–1.18)	66 (33)		0.97 (0.68–1.39)
TNFR1 rs2234649 A>C							
AA	175 (77)	58 (74)	0.56a	1	77 (76)	0.87a	1
AC	45 (20)	16 (21)		1.07	22 (22)		1.11
CC	7 (3)	4 (5)		1.72	2 (2)		0.64
AA	175 (77)	58 (74)	0.62b	1	77 (76)	0.86b	1
AC+CC	52 (23)	20 (26)		0.86 (0.47–1.56)	24 (24)		0.95 (0.54–1.65)
CC	7 (3)	4 (5)	0.40b	1	2 (2)	0.57b	1
AC+AA	220 (97)	74 (95)		1.69 (0.35–6.89)	99 (98)		0.63 (0.06–3.41)
Alleles							
A	395 (87)	132 (85)	0.45b	1	176 (87)	0.94b	1
C	59 (13)	24 (15)		0.82 (0.49–1.37)	26 (13)		1.01 (0.62–1.67)

SIL squamous intraepithelial lesion, LSIL, Low SIL; HSIL, High SIL; OR, odds ratio.

^a^*p* value, two sided, from χ^2^ test for trend.

^b^*p* value, two sided, from χ^2^ test.

^*^p value comparing LSIL with control.

^**^p value comparing HSIL with control.

TNFR1 rs767455 A>G and *TNFR1 rs2234649 A>C* had similar haplotype distribution in SIL patients. The haplotypes *rs767455A*/*rs2234649A* and *rs767455G/rs2234649A* were more frequently observed in both groups. We observed a moderate linkage disequilibrium (LD) between *rs767455 and rs2234649* in SIL patients (D’ = 0.489, *p* = 0.007) and in control (D’ = 0.521, *p* = 0.0031) group.

We determined whether patients in HSIL group, presenting cervical intraepithelial neoplasia (CIN) grade II and III, had different frequencies in the TNFR1 genotypes. No statistical differences were observed between these two groups and when compared with LSIL and control groups (data not shown).

### TNFR1 expressing cells in cervical lesions

**Figure 1:**
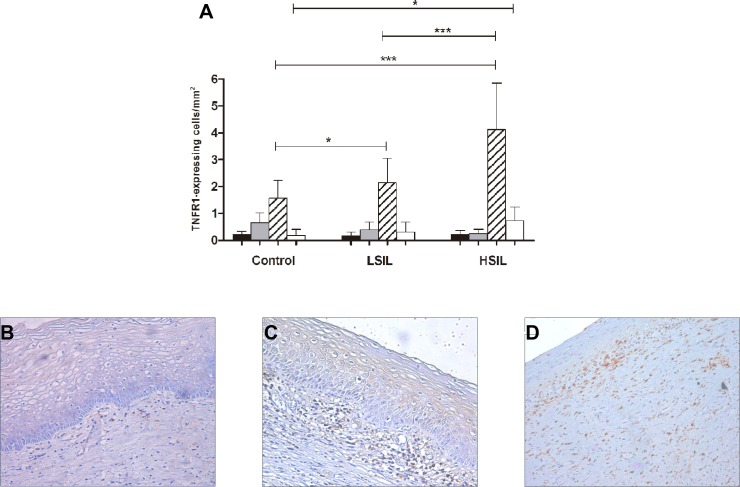
Distribution (**A**) and immunohistochemistry (**B–D**) evaluation of TNFR1 expressing cells in uterine cervix from healthy control (B), low (C) and high (D) squamous intraepithelial lesions. Staining distribution was identified in epithelium (black bars), basal layer of epithelium (light gray bars), stroma (lined bars) and perivascular area (white bars). Two-way Anova, after Bonferroni correction test *p < 0.05; **p < 0.001. Magnification of 200×.

To evaluate the TNFR1 protein expression and its *in situ* cervical location, we selected one-hundred and three samples from 25 healthy control, 38 LSIL and 40 HSIL volunteers. TNFR1-expressing cells were barely expressed in keratinocytes and inflammatory cells in the epithelium and in the basal layer of epithelium in all groups. However, a gradated increase in TNFR1-expressing cells with inflammatory cells morphology was observed in the stroma area in all SIL, mainly in HSIL group when compared to LSIL (1.9-fold, *p* < 0.001) and control groups (2.6-fold, *p* < 0.001). In perivascular area, there was a significant increase in TNFR1 expressing cells in the HSIL group when compared to control group (*p* < 0.05) (Figure 1). These results may indicate an enhancement of inflammatory cells frequency as a response to cervical lesions severity in an attempt to prevent cancer cell invasion. As patients with HSIL were more likely to use tobacco, we tested whether TNFR1 expression was related to this social condition. Our results showed no association between tobacco users and the frequency of cervical TNFR1-expression (data not shown).

Even in the absence of statistical differences in patients with CIN II and CIN III in the TNFR1 gene polymorphisms, we tested whether TNFR1 expressing cells were differently presented in these patients. However, neither TNFR1 gene polymorphisms nor TNFR1-expressing cells were able to distinguish these cervical lesions. Furthermore, both CIN II and CIN III groups were different when compared to CIN I and control groups, showing no overall differences regard to SIL or CIN classification.

### Association of TNFR1 expression and *TNFR1 rs767455A>G* and *TNFR1 rs2234649A>C* SNPs in cervical lesions

**Figure 2:**
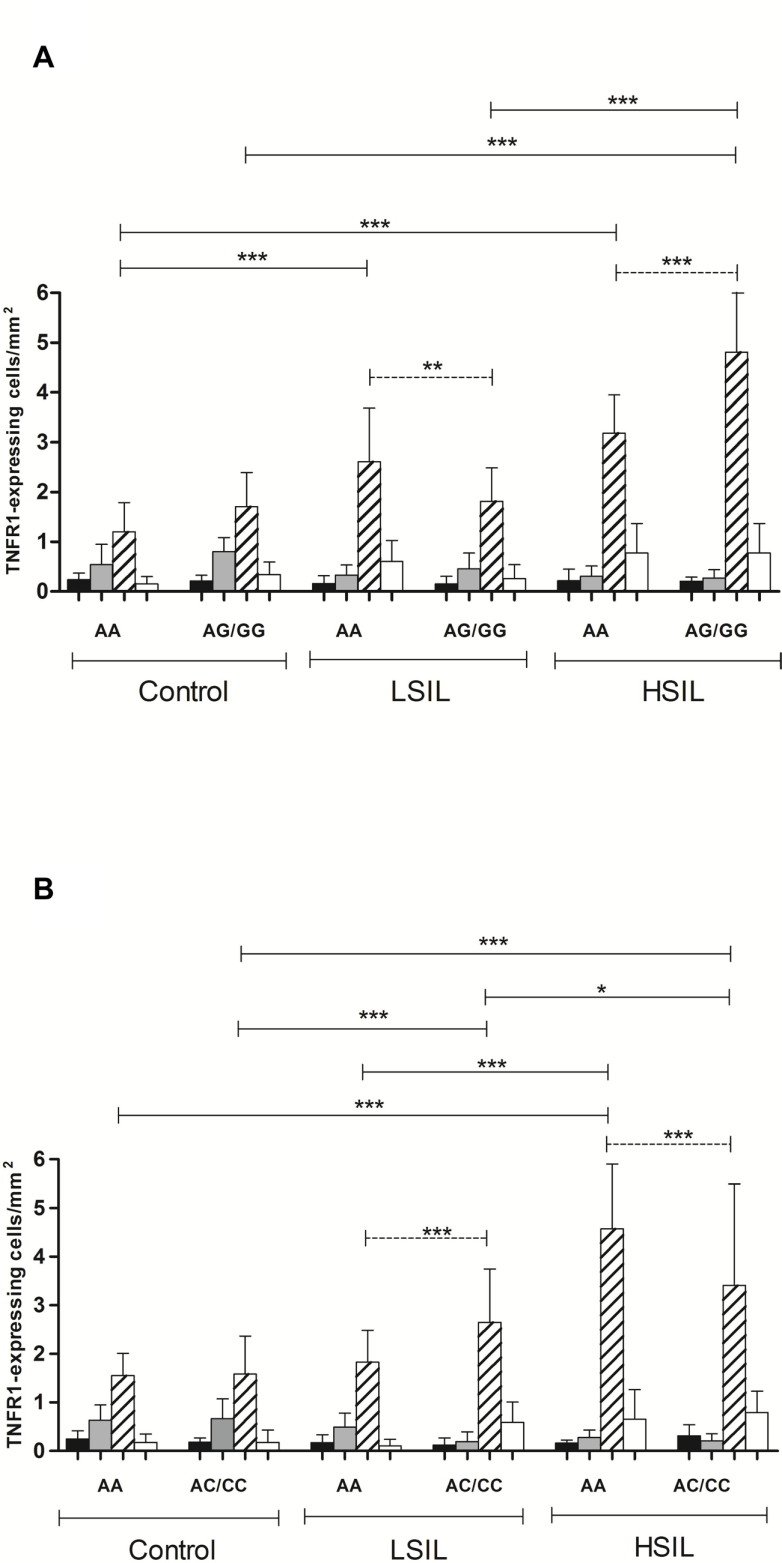
Distribution of TNFR1 expressing cells in uterine cervix from low (LSIL) and high (HSIL) squamous intraepithelial lesions and control groups carrying TNFR1 rs767455 A>G (**A**) and TNFR1 rs2234649 A>C (**B**) polymorphism in epithelium (black bars), basal layer of epithelium (light gray bars), stroma (lined bars) and perivascular area (white bars). Dotted lines indicate intragroups analysis and simple lines indicate intergroups analysis. Two-way Anova, after Bonferroni correction test *p < 0.05; **p < 0.01; ***p < 0.001.

Analyzes were performed to evaluate the association between the *TNFR1 rs767455* and *rs2234649* polymorphisms with cervical TNFR1 protein expression. As *TNFR1rs767455GG* and *TNFR1rs2234649 CC* genotypes showed low frequency in our population, for further analysis the *rs767455AG* and GG and *rs2234649AC* and CC genotypes were combined. TNFR1-expressing cells were not influenced by TNFR1 rs767455A>G and TNFR1 rs2234649A>C genotypes in epithelium, basal layer of epithelium and perivascular area (<1 cell/mm^2)^, in all study groups, as shown in Figure 2A and 2B. However, in stroma area, TNFR1 rs767455AA genotype carriers presented lower distribution of TNFR1-expressing cells when compared to TNFR1 rs767455AG/GG in HSIL group (*p* < 0.001). Interestingly, the TNFR1-expressing cells numbers were higher in TNFR1 rs767455A>A genotype only in LSIL group (*p* < 0.01). Intergroup analysis showed an increased TNFR1 expression in *TNFR1 rs767455AA* carries in HSIL (2.6-fold, *p* < 0.001) and LSIL (2.2-fold, *p* < 0.001) patients compared to control group. In *TNFR1rs767455AG/GG* carriers, TNFR1-expressing cells numbers were 2.8 fold higher in HSIL subgroup when compared to control group (*p* < 0.001) and 2.7 fold higher when compared to LSIL group (*p* < 0.001). No significant difference was found between LSIL and control subgroups (Figure 2A).

The *TNFR1 rs2234649*A>C did not influence the distribution of TNFR1-expressing cervical cells in control group. However, when *TNFR1 rs2234649*AA genotype was compared to *rs2234649AC/CC* genotypes, there was a predominant TNFR1-expressing cells numbers in *rs2234649AA* carries in HSIL (*p* = 0.001) and AC/CC genotypes in LSIL (*p* < 0.001) groups. In intergroup analysis, *TNFR1 rs2234649AA* homozygote carriers showed increased TNFR1-expressing inflammatory cells in HSIL when compared to control (3.1-fold, *p* < 0.001), and LSIL groups (2.5-fold, *p* < 0.001). No significant difference was found between LSIL and control groups. Regard TNFR1 *rs2234649* AC/CC genotypes, an increase in TNFR1-expressing cells was 1.6 fold higher in LSIL group compared to control group (*p* < 0.001); 2.1-fold higher in HSIL compared to control group (*p* < 0.001) and 1.3-fold higher when compared LSIL (*p* < 0.05) (Figure 2B).

Even in the absence of statistical differences in patients with CIN II and CIN III in the *TNFR1* gene polymorphisms, we tested whether TNFR1 expressing cells were differently present in these patients. Unfortunately, neither TNFR1 gene polymorphisms nor TNFR1-expressing cell were able to distinguish CIN-II and CIN-III cervical lesions.

These data indicate that pre-malignant lesions induce an inflammatory process with increasing TNFR1-expressing cells distribution. Besides, *TNFR1 rs767455* AG/GG and *TNFR1 rs2234649* AA genotypes might be associated with the development of high grade cervical lesions.

## DISCUSSION

Cervical cancer is one of the most common gynecological malignancies in Brazilian population and more than 50% of young adults are infected. Most of cervical lesions regress spontaneously, indicating that only HPV infection is not sufficient for inducing carcinogenesis. Other factors are necessary for inducing carcinogenic process, such as environmental, behavioral and genetic factors. This study investigated the *TNFR1 rs767455 A>G* and *TNFR1 rs2234649 A>C* SNPs in SIL development risk.

Demographic data analyses showed higher mean in age in SIL, especially in HSIL group, when compared to control group. It has been described that older women may develop more severe cervical lesions. POP-Brasil surveillance reported a higher HPV-DNA persistence in Brazilian women aged 16-26 years, enhancing the probability to high-grade lesions progression [6] [preliminary results available at http://portalms.saude.gov.br/noticias/agencia-saude/42003-estudo-apresenta-dados-nacionais-de-prevalencia-da-infeccao-pelo-hpv], and similar observation was described in Japanese women [30]. HPV can be transmitted in first sexual intercourse before 20 years old, persisting between 25-30 years, reaching a peak after 55 years old [31]. In addition, even if the majority of HPV infections are transient, cofactors may affect the risk of lesions development and persistence, including women age and behavior [32].

We observed that tobacco use has been associated with increased risk of high-grade lesions. The nicotine and its metabolite, cotinine, were found increased 4-fold in cervical mucus from healthy women smokers and 40-fold in women with SIL [33]. However, repair defects related to carcinogenesis and DNA damage were observed in smoker’s cervical tissues. Benzo[a]pyrene (BaP), identified as prime carcinogen in cigarette smoke, was detected in cervical tissue, and DNA adducts were present in smokers twice as often as in noncurrent smokers [34]. Besides, nicotine and cotinine were associated with Langerhans cells reduction in uterine cervix from smokers, especially in HSIL women [35]. It has been suggested that the reduction of the antigen presenting cells population would be a responsible factor for the decrease in local immune response and SIL development [36]. Besides the high frequency of smokers in SIL patients, the TNFR1 expression in cervical lesion was not different from those non-smokers, indicating that TNFR1 expression is not influenced by nicotine itself.

In the present study, no association was found between *TNFR1 rs2234649* and *rs767455* SNPs with SIL in any genetic model tested. To our knowledge, there are no reports evaluating the *TNFR1* polymorphisms in cervical cancer and/or HPV-related cervical lesions.

Regard to other cancers types, few investigations have analyzed the role *TNFR1 rs2234649* or *rs767455* SNPs in cancer development. No association was found between *TNFR1 rs2234649* and gastric mucosa-associated lymphoid tissue lymphoma [24], and in myelopathy associated to HTLV-1 infection [37]. However, controversial results were found in *TNFR1 rs767455* SNPs, where no association was described in esophageal squamous cell carcinoma [23], while a significant increased risk for breast cancer [38, 39] and odontogenic keratocystic tumor [40] were observed. Moreover, others *TNFR1 SNPs* have been described in different cancer types. *TNFR1 rs4149570 G>T* SNP have been associated with loss of heterozygosity in hepatocellular carcinoma, indicating that this SNP is susceptible to cancer genetic alterations [25]. In non-small cell lung cancer, this SNP has been associated with poor survival in patients under these conditions [26]. Wu *et al.* [2011] observed that patients carrying the wild-type allele G of *rs4149579 G/T* SNP had reduced risk for gastroesophageal reflux disease, which is a risk factor for esophageal adenocarcinoma [27]. In oral carcinoma, TT genotype of this SNP conferred a protective character on the cancer development [28].

We further evaluated the distribution of TNFR1-expressing cells in SIL and its association with cervical lesion development. We observed a rare cell distribution (<1.0 cells / mm^2^) in epithelium and in the basal layer of the epithelium in all groups. However, our study showed a progressive increase of TNFR1-expressing inflammatory cells distribution nearby the lesion, in the stroma area according to lesion severity. It is noteworthy that our study is the first to correlate the distribution of TNFR1-expressing cells in HPV-associated cervical lesions and its progression to pre-malignant lesions.

TNFR1 protein expression, in its monomeric form, is constitutively expressed in all human tissues and appears to be altered by cytokines, especially in the epidermis [41]. The molecule trimerization leads to activation and binding in soluble TNF-α. TNFR1 expression has been observed in granulocytes, but it is low expressed in lamina propria lymphocytes [42]. In epithelial ovarian cancer, the TNFR1 expression was observed in the epithelial cell cytoplasm in both cancer and benign ovarian lesions, showing no association with the onset, or disease stage [43]. A study conducted in China by Ma *et al.* (2015) evaluated both TNFR1 and TNFR2 receptors in hypopharyngeal squamous cell carcinoma (HPSCC). They observed expression of these receptors in all specimens of HPSCC. However, when performed a ratio analysis of these two receptors expression, it was observed that TNFR2 expression is negatively correlated to TNFR1 expression, suggesting that the TNFR1 can dominate the interactions between the two receptors and, therefore, the clinical HPSCC outcome [44].

HPV infection can influence apoptotic signals transduction generated by TNF-α interaction with its receptor. Filippova *et al.* (2002) observed that HPV16-E6 oncoprotein does not interfere with protein expression, but inhibits the TNFR1 and TRADD interaction [14]. Likewise, E6 oncoprotein binds to the TNFR1 receptor, interfering with the TNFα-induced pro-apoptotic signaling, suggesting impairment in death-inducing signaling complex (DISC) through caspase cascade activation, such as caspase-8 [45].

In previous work, we demonstrated the expression of TNF-α and higher inflammatory cells distribution, both CD4^+^ and CD8^+^ cells T cells, in the uterine cervix increasing according to the lesion severity [8]. Similarly, Alves *et al.* (2010) observed high numbers of CD4^+^ and CD8^+^ T lymphocytes in HSIL and cervical carcinoma [46]. There are few reports in the literature addressing TNFR1 expression in CD4^+^ T cells in cervical pre-malignant lesions or associated with carcinogenesis. However, the involvement of CD4^+^ T cells and its relationship with TNFR1 have been demonstrated in other chronic inflammatory diseases such as rheumatoid arthritis (RA) [47]. The cellular migration can be related to the TNFR1 expression, since this receptor is expressed on a fraction of CD4^+^ T in RA patients, but not in control group; this migration is dependent on the TNF-α concentration gradient *ex vivo,* and blocking of TNF-α or TNFR1 expression resulted in abrogation of CD4^+^ T cells migration in synovial tissue [47]. TNF-α markedly promotes tumor lymphangiogenesis and lymphatic metastasis through TNF-α-TNFR1 signaling pathway, activating inflammatory macrophages and tumor-associated macrophages (TAMs) to produce high levels of VEGF-C. TNFR1 mediates TNF-α-induced tumor lymphangiogenesis and metastasis by modulating VEGF-C-VEGFR3 signaling [48].

The TNFR1 expressing cells in cervical stroma in SIL patients were composed primarily of mononuclear cells, including mainly macrophages, lymphocytes, rarely Langerhans cells and monocytes, as part of inflammatory cells pool expressing TNFR1. In previous study, we demonstrated that rare monocytes were present in cervical lesions [8], but it may be associated with intra-tumoral macrophages origin and activation. Further studies may address the role of TNFRs regarding cell migration, activation and their apoptotic functions in cervical cancer.

We further evaluated the possible correlation between *TNFR1* polymorphisms and expression in SIL patients. Regarding the *rs767455A>G*, TNFR1-expressing cells distribution was higher in *AG/GG* carriers, while in *rs2234649A>C*, the distribution was greater in women carrying in the *AA* genotype. There are no reports in the literature associating *in situ* TNFR1 expression and its SNPs in the uterine cervix and thus, we should infer that the TNFR1 expression can be negatively modulated by HPV infection and TNFR1 biomarkers are discriminatory between normal and SIL cervix and may be used as an indicator for the cervical precancerous lesions progression.

## MATERIALS AND METHODS

### Study subjects and tissue samples

Four hundred and six non pregnant HIV-1 seronegative women were enrolled in this study from November, 2008 to October, 2010, aged 18 years older. Patients were included at Fernandes Figueira Woman, Child, and Adolescent National Institute of Oswaldo Cruz Foundation (IFF/Fiocruz), Rio de Janeiro, Brazil and submitted to a gynecological exam with colposcopy by certified gynecologist. According to the histopathological analysis at the baseline gynecological visit, two groups were defined as having low (LSIL) and high (HSIL) grade squamous intraepithelial lesion (SIL) [49]. Patients received free appropriated treatment [50]. Healthy control group (*n* = 227) with no proven cervical lesions and who were genetically unrelated to the SIL cases were recruited from three Clinical sites at Rio de Janeiro, RJ, Brazil: INI/Fiocruz, Pedro Ernesto University Hospital (HUPE-UERJ) and the Américo Piquet Carneiro Polyclinic at State University of Rio de Janeiro (UERJ). In a previous work, samples of the case group showed high positivity for the HPV pool (HPV 1, 6, 11, 16, 18 and 31) (96.3%) and HPV 16 (77.5%) immunostaining [51]. The control group was previously selected [52] who attended the Pedro Ernesto University Hospital (HUPE-UERJ) and the Américo Piquet Carneiro Polyclinic to get routine pelvic exam and Pap smear test, with normal cytology results. Women taking immunosuppressants, suffering from autoimmune diseases or cancer, hysterectomized, virgin, and those who did not tolerate the gynecological exam were excluded from this study. In the control group, women with a history of cervical injury were also excluded. Each participant answered questions regarding classic risk factors for cervical cancer and sociodemographic characteristics, including skin color/ethnicity, and signed an informed consent form. A standardized questionnaire including social and demographic data was applied. The study was conducted following the Declaration of Helsinki set of ethical principles regarding human experimentation. Written consent was obtained from all volunteers and the protocol approved by the Institutional Ethical Review Board from INI/Fiocruz, IFF/Fiocruz and UERJ with the number 0001.0.009.000-05.

### Histopathologic examination

Paraffin-embedded cervical tissues were processed by conventional histopathology and routinely stained with hematoxylin-eosin (HE). Premalignant cervical lesions were classified as LSIL and HSIL, characterized by the stage of epithelial differentiation and maturation by a certified pathologist. The control group was composed of cervical biopsies from hysterectomized women, without HPV-related lesions.

### DNA extraction and polymerase chain reaction-restriction fragment length polymorphism [PCR-RFLP] analysis

The genomic DNA was isolated with the QIAGEN DNA Blood Mini Kit (Qiagen, Valencia, CA) according to the manufacturer’s instructions. The quality of extracted DNA was checked by electrophoresis in a 1% agarose gel where an ethidium bromide stained single band was visualized under ultra-violet light. Subsequently, DNA fragments containing the *TNFR1* rs767455 (*TNFR1+36A/G*) and rs2234649 (*TNFR1-383A/C)* SNPs were amplified by PCR.

For both polymorphisms, the reaction mixture was carried out in a total volume of 25 µl, containing 100 ng genomic DNA; 40.8 nmol of each primer *TNFR1* rs767455: FW 5′-GAGCCCAAATGGGGGAGTGAGAGG-3′; RV 5′-ACCAGGCCCGGGCAGGAGAG-3′ [31]. The Primer-BLAST tool from the NCBI database was used to design the specific primer of *TNFR1* rs2234649 through its reference sequence (RefSeq) NG_007506.1: FW 5′-TTATTGCCCCTTGGTGTTTGGTTG-3′; RV 5′-TTGTGACGGAGTGAGAAGGGGAGG-3′ (Invitrogen^®^); 100 mM of dNTP (Applied Biosystem^®^); 25 mM of MgCl_2_; 2.5 µl of 5x buffer (Promega); 5U/µl of Taq DNA polymerase (Invitrogen^®^). PCR was performed in Thermal Cycler (Applied Biosystems). The cycles used were: 95° C (5 minutes), 35 cycles of 95° C (1 minute), 73°C (1 minute) and 72° C (1 minute) followed by 72° C (5 minutes) and 4°C (temperature of completion).

The resultant PCR products were digested using the restriction endonucleases *MspaA1I (rs767455) and BglII (rs2234649)* at 37° C for 16 hours. The digested product was separated on a 3% agarose (Amersham Biosciences AB) gel stained with ethidium bromide. The resulting 183-bp fragment of *TNFR1 rs767455* digested with *MspaA1I* produced a single 183bp fragment (A allele) or fragments of 108 and 75 bp (G allele). *TNFR1 rs2234649* generated a 370 bp fragment and digestion with *BglII* gave rise to a single 370bp fragment (A allele) or fragments of 240 and 130 bp (C allele).

### *In situ* expression of TNFR1 from cervical lesions

Serial paraffin-embedded tissue sections (3 µm) were fixed in silane-coated slides. To determine the *in situ* expression of TNFR1, immunohistochemistry (IHC) technique was performed according to the REVEAL Biotin-Free Polyvalent HRP manufacturer’s instructions (Spring, CA, USA). Sections were incubated overnight at 4°C with specific antibody against TNFR1 (1:100, Santa Cruz, Texas, USA). Positive stained cells were counted in twenty fields (400x) in the epithelium, basal layer of epithelium, stroma and perivascular areas of uterine cervix. Counts were performed using a grid (1 cm^2^ divided into 10 × 10 mm^2^) by two different observers.

To ensure reproducibility, all cases were examined by two observers in order to derive concordance.

### Statistical analysis

The Hardy–Weinberg equilibrium was tested comparing observed and expected genotype frequencies in the two studied groups (SIL and controls) using the χ^2^ test. Differences in genotype, allele and haplotype combination frequencies between the group and subgroups of cases (patients with LSIL and HSIL) and controls or between the subgroups of cases were evaluated by a 2 × 2 χ^2^ contingency table or Fisher’s exact test (in case of samples lower than 5) and a 3 × 2 c^2^ for trend contingency table to evaluate the dominant model by Epi-Info Software. Three types of genetic models (dominant, co-dominant and recessive) were used for the association analysis of *TNFR1* polymorphisms with susceptibility to cervical neoplasia or severity of cervical lesion. Odds ratio (OR) and 95% confidence interval (CI) of each genotype or allele compared to the reference genotype and allele were calculated to quantify the magnitude of the association. Uni and multivariate logistic regression analysis were used to define the significance of the genetic predictor, with OR and 95% CI of SIL patients, and LSIL and HSIL subgroups versus control group, using clinical and environmental data, such as age, ethnicity, tobacco use, age of the first sexual intercourse, menarche and number of pregnancies, using SNPStats http://bioinfo.iconcologia.net/SNPstats software. Further, the significant variables were also evaluated using Bonferroni correction, if applicable. Haplotype frequency and Pairwise Linkage disequilibrium (D’) were determined using SNPStats. TNFR1 mean of expressing cells was compared by two-way ANOVA test among SIL and control groups, and among LSIL and HSIL subgroups. Significant variables were also corrected by Bonferroni post test, using GraphPad Prism 5. Significance was define as *p* value < 0.05.
